# Dual task interference on early perceptual processing

**DOI:** 10.3758/s13414-020-02158-0

**Published:** 2020-10-20

**Authors:** Justin Duncan, Amélie Roberge, Ulysse Fortier-Gauthier, Daniel Fiset, Caroline Blais, Benoit Brisson

**Affiliations:** 1 Département de psychologie, Université de Fribourg, Fribourg, Switzerland; 2 Département de Psychoéducation et de Psychologie, Université du Québec en Outaouais, Gatineau, Québec Canada; 3 Département de Psychologie, Université du Québec à Trois-Rivières, Trois-Rivières, Québec Canada

**Keywords:** Psychological refractory period, Dual task, Central attention, Visual-spatial attention, Visual working memory, Task switching, Attentional blink

## Abstract

When two tasks, Task 1 and Task 2, are conducted in close temporal proximity and a separate speeded response is required for each target (T1 and T2), T2 report performance decreases as a function of its temporal proximity to T1. This so-called psychological refractory period (PRP) effect on T2 processing is largely assumed to reflect interference from T1 response selection on T2 response selection. However, interference on early perceptual processing of T2 has been observed in a modified paradigm, which required changes in visual-spatial attention, sensory modality, task modality, and response modality across targets. The goal of the present study was to investigate the possibility of early perceptual interference by systematically and iteratively removing each of these possible non perceptual confounds, in a series of four experiments. To assess T2 visual memory consolidation success, T2 was presented for a varying duration and immediately masked. T2 report accuracy, which was taken as a measure of perceptual—encoding or consolidation—success, decreased across all experimental control conditions as T1–T2 onset proximity increased. We argue that our results, in light of previous studies, show that central processing of a first target, responsible for the classical PRP effect, also interferes with early perceptual processing of a second target. We end with a discussion of broader implications for psychological refractory period and attentional blink effects.

**Electronic supplementary material:**

The online version of this article (10.3758/s13414-020-02158-0) contains supplementary material, which is available to authorized users.

Contemporary life frequently requires individuals to concurrently process multiple stimuli or carry more than one task at a time. Yet despite the impressive flexibility and capacity of the human mind for adaptation, dedication to multiple overlapping tasks (i.e., multitasking) comes at a cost. This cost can be studied in a laboratory setting using highly useful experimental procedures: the psychological refractory period (PRP; Telford, 1931) and attentional blink (AB; Raymond, Shapiro, & Arnell, 1992) paradigms. Though the present study mainly focuses on the former, we will later see how it may provide useful insight on the link between PRP and AB multitasking deficits.

## Psychological refractory period—Paradigm

Dual task PRP paradigms involve having participants perform a sequence of two separate speeded tasks, Task 1 (e.g., auditory discrimination of a first target; T1) and Task 2 (e.g., visual categorization of a second target; T2). The critical manipulation is the degree of overlap between processing stages of both tasks, which is achieved by varying T1–T2 stimulus-onset asynchrony (SOA). In so doing, researchers manage the extent to which processing of the prioritized T1 can interfere with that of the nonprioritized T2. Classically, increasing overlap (by reducing SOA) between T1 and T2 processing stages will cause a progressive increase in response times to T2 (RT2). However, when T2 is backward masked—that is, quickly replaced by a task-irrelevant stimulus—report accuracy of T2 is also impaired (Brisson & Jolicoeur, 2007c; Pashler, 1991). The general decrease in T2 performance is what is referred to as the PRP effect (Pashler, 1994; Welford, 1952). Despite individual differences in magnitude, the PRP effect is highly robust. As such, one can easily imagine the adverse consequences of carrying speeded concurrent tasks when transposed to naturalistic activities that require quick reactions, such as, for example, the sudden need to brake or swerve to avoid hitting a previously hidden obstacle or person while driving a car (Levy, Pashler, & Boer, 2006).

## Psychological refractory period—Models

Several models have been proposed to account for the PRP effect (see, for review, Wu & Liu, 2008). However, when instructions emphasize prioritization of T1, and also a quick and accurate response to both T1 and T2, the central bottleneck model, a specific instance of another prominent model (central capacity sharing; Navon & Miller, 2002; Tombu & Jolicoeur, 2003), appears to offer the most parsimonious account (Pashler, 1984, 1994; Pashler & Johnston, 1989). According to the central bottleneck model, the PRP effect has its source at central stages—that is, between (early) perceptual and (late) response execution stages—hence, the *central* bottleneck (McCann & Johnston, 1992). Specifically, the model posits that response selection—the process of pairing a stimulus to a behavioral response—is the critical central process responsible for the bottleneck. As such, it is when response selection stages of T1 and T2 overlap that central interference on T2 processing (i.e., the PRP) occurs. On the other hand, prebottleneck and postbottleneck processing are assumed to be carried out without central processing resources.

These basic assumptions of the central bottleneck are mostly also those of the central capacity-sharing model. However, the main differentiating factor between the two is that the former assumes an all-or-none bottleneck, whereby all available central resources are first allocated to T1 and then switched to T2 once central T1 processing is complete. On the other hand, the central capacity-sharing model is not all-or-none, but instead leaves space for strategic sharing of central resources, depending on instructions. For instance, at short SOAs, it would be possible to allocate 70% of resources to central T1 processing while simultaneously allocating 30% of central resources on central T2 processing. However, it is also possible, according to the central capacity-sharing model, to strategically allocate 100% of resources to central T1 processing and 0% to T2 processing, which is why the central bottleneck model may be considered as an instance of the capacity-sharing model.

## Early PRP interference

An important prediction of these two prominent models—central bottleneck and central capacity-sharing—is that perceptual processing of T2 can be carried out concurrently, without interference from central processing of T1. This prediction has been supported by many behavioral studies using the locus of slack logic (Schweickert, 1978). However, there is mounting evidence that several processes preceding T2 response selection are also sensitive to PRP-induced interference, such as visual short-term memory (vSTM) consolidation, visual-spatial attention, stimulus categorization, and early cortical stages of perceptual encoding (Brisson & Jolicoeur, 2007a, b, c; Janczyk, Augst, & Kunde, 2014; Johnston & McCann, 2006).

A particularly informative electrophysiological study to this effect was conducted by Brisson and Jolicoeur (2007b). In this study, event-related potentials (ERP) were measured while participants performed a modified cross-modal PRP paradigm. A single SOA value was calculated on a participant basis to both maximize T1–T2 overlap in a hard T1 condition and minimize overlap in an easy T1 condition. Accordingly, contrasting easy and hard conditions served as a proxy for the PRP effect, which they then measured across several ERPs: the P3 index of short-term memory updating and conscious access (Donchin, 1981; Donchin and Coles, 1988; Luck, 1998), the N2pc (N2 posterior contralateral) index of visual-spatial attention (Brisson, Robitaille, & Jolicoeur, 2007; Eimer, 1996; Luck & Hillyard, 1994), the SPCN (sustained posterior contralateral negativity) index of vSTM consolidation (Jolicoeur, Brisson, & Robitaille, 2008; Vogel & Machizawa, 2004), and finally, the P1 and N1 indices of visual encoding.

When the visual T2 task required a four-alternative visual discrimination, they observed amplitude decreases across all components. Thus, central processing of the difficult auditory T1 interfered with short-term memory updating (an effect analogous to that found in AB; Vogel, Luck, & Shapiro, 1998), visual-spatial attention, vSTM consolidation and even earlier visual encoding (see also, for similar findings, Brisson & Jolicoeur, 2007a, c; Brisson, Leblanc, & Jolicoeur, 2009; Lien, Croswaite, & Ruthruff, 2011; but see, however, for conflicting results regarding visual-spatial attention, Reimer, Strobach, & Schubert, 2017). Additionally, the SPCN was also delayed, suggesting vSTM consolidation also took longer or started later, thereby making whatever information was encoded more susceptible to decay. In contrast, when the visual T2 task required a simple target detection, interference on short-term memory updating remained manifest, but visual encoding was largely preserved (N2pc and SPCN amplitudes were not analyzed in this case). This mimics the task difficulty manipulation by Jolicoeur (1999) and suggests the effect is not all or none (Roberge, Duncan, Fiset, & Brisson, 2019), but may instead be dose dependent. Processing that does occur—perhaps more coarsely—in spite of PRP interference would suffice to support basic visual detection, but not visual discrimination, which requires more extensive processing.

## Present study

Though these previous results are striking in that they suggest early perceptual encoding is susceptible to central interference, a number of confounding factors preclude an immediate jump to this conclusion. First, the PRP paradigm developed by Brisson and Jolicoeur (2007b) is not exactly typical in that SOA was not parametrically manipulated: though a proxy for PRP was established by contrasting easy and hard T1 conditions, its central processing overlap remains fundamentally indissociable from T1 difficulty. Our first objective was to test these early perceptual effects using a more typical PRP paradigm, whereby SOA was parametrically manipulated and importantly, orthogonal to other task parameters, as in Brisson & Jolicoeur (2007a).

Second, the visual T2 was presented laterally to a central fixation, and thus required a deployment of visual-spatial attention to be adequately processed; and spatial attention, as we have just seen, requires central resources. Third, T2 was always preceded by an auditory (i.e., cross-modal) T1, and T2 processing was thus necessarily preceded by a change in sensory modality. Fourth, T1 and T2 were associated with different tasks, and T2 processing therefore also required task switching. Finally, responses to T1 and T2 were delivered using different hands, and thus, T2 response output required a change in response modality. Hence, any one of these, all necessary to successfully carry T2 processing, could have been the locus of PRP interference observed by Brisson and Jolicoeur (2007b). Our second objective was therefore to test each of these possible causes of PRP interference by systematically eliminating every alternative, one at a time, to verify if central processing of T1, responsible for the PRP effect, interferes with early perceptual processing of T2.

To attain this goal, we conducted a sequence of four iterative behavioral experiments, with each one building upon the last. Experiment 1 was designed to rule out visual-spatial attention as the locus of PRP interference. Experiment 2 was designed to rule out sensory modality switching. Experiment 3 was designed to rule out task switching. And finally, Experiment 4 was designed to rule out response modality switching as the locus of PRP interference. We surmised that if some PRP interference remained after having eliminated all of these putative sources of interference, then we could safely conclude that early perceptual processing is also subjected to PRP interference.

We favored a behavioral approach in which a mask was introduced shortly after T2 onset to target visual encoding (Jolicoeur, 1999), thereby dissociating early effects from later central and motor effects. Given that visual masking resets retinal input and precludes further stimulus encoding in visual working memory (Vogel, Woodman, & Luck, 2006), T2 report accuracy was selected as the dependent variable over RT2, as it would be a better indicator of stimulus encoding success (e.g., Pashler, 1991). If processing of T1 interferes with early perceptual processing of T2, either affecting perceptual encoding or visual short-term memory consolidation, then a decline in T2 report accuracy will appear as SOA is reduced, even after spatial attention, sensory modality switching, task switching, and response modality switching have all been accounted for.

To anticipate the results, a decline of T2 accuracy was observed in each experiment. As we shall see, a noteworthy part of our study is that PRP task variants became, as Experiments progressed, more similar to an AB task, whereby report accuracy declines as two targets, embedded in a rapid visual presentation of distractors, are presented in greater temporal proximity from one another. Although the PRP effect and the AB deficit have mostly been considered separately, the present results favor an integrative approach, which may ultimately help bridge the two phenomena and possibly lead to a unified understanding of multitasking deficits.

## Experiment 1: Visual-spatial attention

Experiment 1 was designed to investigate whether shifting of visual-spatial attention could be the source of PRP interference observed by Brisson and Jolicoeur (2007a, b, c) by manipulating the position of T2 (peripheral vs. central). A task similar to those devised by the same authors was employed. Specifically, participants performed a PRP paradigm, whereby the pitch of an auditory T1 (200 Hz, 430 Hz, 926 Hz, or 2000 Hz) had to be categorized, and then, following a variable SOA (300 ms, 650 ms, or 1,000 ms), the gap on a visual T2 square (left, right, top, or bottom) was to be localized.

For one half of the sample (peripheral condition), the T2 square was presented laterally to one hemifield and accompanied by a distractor square in the opposite hemifield. For the other half of the sample (central condition), the T2 square was presented in central vision, without any distractor. Based on the results of Brisson and Jolicoeur (2007a, b, c), a negative effect of shorter SOA on T2 accuracy was expected in the peripheral condition. If, however, a similar effect was to be observed in the central group, then this would mean that visual-spatial attention shifts cannot on their own account for interference observed in T2 at short SOA.

### Method

#### Participants

Thirty-one French-speaking volunteers participated in this experiment for financial compensation. Seven participants were excluded (see Analyses, below). Thus, our final sample consisted of 24 participants (13 women), ages 19–32 years (*M* = 22.2, *SD* = 2.9), divided into two groups (central/peripheral condition; see Design and Procedure section).

PRP effect size is typically large and reliable. It can thus be obtained with fairly small samples (e.g., fewer than 10 in Brisson & Jolicoeur, 2007a). For example, expecting an admittedly conservative ${\eta}_p^2$ equal to 0.4 with alpha equal to 0.05, a sample of *N* = 8 would achieve power equal to 0.97—that is, there would be a 97% probability of detecting a true PRP effect according to a G*Power analysis (Faul, Erdfelder, Buchner, & Lang, 2009). To accommodate possible reductions of PRP effect sizes as a result of experimentally removing possible PRP loci of interference, we opted for a modestly larger sample size throughout this study. The current and following experiments are designed to detect PRP effect sizes of ${\eta}_p^2$ equal to 0.25 with power ranging from 0.9 (Experiments 1 and 4) to 0.98 (Experiments 2 and 3).

All subjects were neurologically intact and reported having normal or corrected-to-normal visual acuity and color vision, as well as normal audition. Written consent was obtained from each participant at the beginning of the experiment. The procedure complied with the Declaration of Helsinki and was approved by the Research Ethics Committee at Université du Québec à Trois-Rivières (UQTR-REC).

#### Stimuli and apparatus

Task 1 auditory targets were four pure tones of either 200 Hz, 430 Hz, 926 Hz, or 2000 Hz, emitted simultaneously by two loudspeakers placed on either side of the computer screen. Task 2 visual targets were red and green squares (1° × 1°) with an opening (0.33°) on one of their four faces, overlaid on a black background. T2 visual masks were white squares of the same size with openings on all four sides, overlaid on a black background. The experiment was programmed in E-Prime 2.0 and ran on a 16-in. CRT computer monitor, with a 1,024 × 768 screen resolution and a 60-Hz refresh rate. Participants sat 67 cm from the monitor.

#### Design and procedure

A mixed design was employed with two within-subjects factors and one between-subjects factor. The within-subjects factors were SOA (300 ms, 650 ms, and 1,000 ms), and T2 duration (133 ms, 150 ms, 166 ms, and 183 ms). All within-subjects conditions were randomly intermixed across trials within each block. The between-subjects factor was T2 position: one group of participants (*N* = 12) was presented with peripheral targets (3.5° to the left or right of the central fixation point), and the other group (*N* = 12) was presented with central targets (i.e., at fixation).

In the peripheral condition, red and green squares were presented simultaneously on either side of central fixation. Target/distractor status of each color was counterbalanced across participants: Half (*N* = 6) treated red squares as targets, and the other half treated green squares as targets. No two openings from squares of a target–distractor pair shared the same location. In the central condition, only a central red or green colored target square was presented (i.e., no distractor square). Half of participants (*N* = 6) were presented red squares, and the other half were presented green squares.

The experiment began with the presentation of written instructions on-screen. Then, each tone was presented twice in sequence, from low to high frequency, for familiarization. Participants then performed a total of 80 practice trials: 32 single T1 trials, 16 single T2 trials, and 32 dual task T1–T2 trials. The experimental task itself consisted of 12 blocks each comprising 32 trials (384 trials total).

**Fig. 1:**
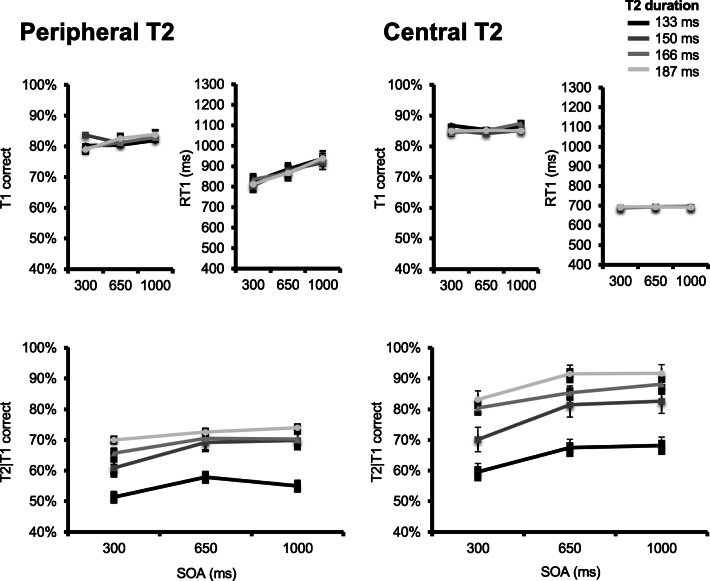
Illustration of a trial sequence from Experiment 1. T1 was presented for 100 ms. It consisted of an auditory tone (200, 430, 926, or 2000 Hz), which participants had to identify. After either a 300, 650, or 1,000 ms stimulus-onset asynchrony (SOA), T2, a colored square with a gap in one side, was presented until a mask (gray square with four gaps), replaced it 133, 150, 166, or 183 ms later, and for 67, 50, 34, or 17 ms. T2 was presented centrally (at fixation), or peripherally (3.5° left/right of fixation) accompanied by a distractor (3.5° right/left of fixation), a differently colored square with a gap in a different side. Participants were asked to localize the target gap once they had given their response to T1

Figure 1 illustrates the course of a trial, which was initiated by pressing the “F” and “J” keys simultaneously with the left and right index fingers, respectively. This led to a blank screen containing only a central fixation point that remained on-screen for a variable interval (between 300 and 500 ms, pseudorandomly chosen on a trial basis). After this interval, the auditory T1 tone was emitted (100 ms), and immediately followed by a variable SOA (300, 650, or 1,000 ms). Then, the visual T2 was presented (133, 150, 166, or 183 ms), and immediately tailed by a visual mask (67, 50, 34, or 17 ms, respectively), such that their combined duration was always 200 ms. T2 stimulus and mask duration was manipulated this way in order to avoid floor/ceiling effects. The sequence terminated when the visual mask was replaced by a blank screen containing only the central fixation, that remained on-screen until participants responded.

Participants were required to make two separate four-choice speeded responses. They first identified the pitch of the auditory T1 tone with their left hand, pressing on the appropriate key: “A” (pinky; 200 Hz), “S” (ring; 430 Hz), “D” (middle; 926 Hz), and “F” (index; 2,000 Hz). Then, they indicated the location of the gap in the colored T2 square with their right hand, again pressing on the appropriate key: “J” (index; left), “K” (middle; bottom), “L” (ring; top), and “;” (pinky; right). Instructions emphasized that participants fixate the central point throughout each trial, and also stressed the importance of responding as quickly and accurately as possible to T1, as soon as T1 was presented, and also responding as quickly and accurately as possible to T2, as soon as T2 was presented.

Following 1,250 to 1,750 ms after a response for each task was registered, the central fixation point was replaced by visual feedback, which took the form of a “+” (correct) or “−” (incorrect), left (for T1) or right (for T2) of central fixation point. Feedback remained visible until the next trial was initiated.

#### Analyses

Our analyses focused on individual mean T1 accuracy, RT1, and T2 accuracy. In addition, for this latest measure, only trials with correct responses to T1 were considered; that is, we measured T2 accuracy given a correct answer to T1 (henceforth, T2|T1).

Experiment 1 mean T1 accuracy, RT1, and T2|T1 accuracy were each analyzed in SPSS 23 (IBM corp.) and jamovi software, using a repeated-measures analysis of variance (ANOVA), with two within-subjects factors (SOA [300, 650, and 1,000 ms]; T2 duration [133, 150, 166, and 183 ms]), and one between-subjects factor (T2 position [peripheral T2, and central T2]). A Greenhouse–Geisser correction was applied when the sphericity assumption was violated.

Participants with (1) mean RT1 above two standard deviations versus group mean (*N* = 4); (2) with mean T1 accuracy lower than 60% (*N* = 2); or (3) with mean T2|T1 accuracy lower than 30% (*N* = 1) were excluded^1^ from the initial data set (i.e., total of seven). Though this may be a high proportion of the sample, setting these criteria was essential to ensure that (1) participants did not simply postpone T1 processing after T2 presentation, (2) a sufficiently large percentage of trials (i.e., 60%) was available for analysis of T2|T1 correct responses, and (3) T2|T1 correct response accuracy was above chance level.

### Results

#### T1 performance

##### T1 accuracy

**Fig. 2:**
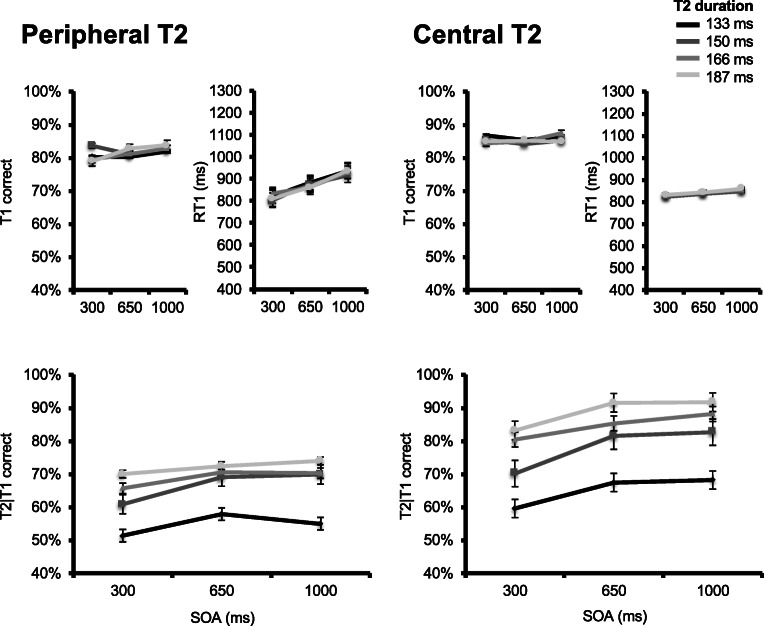
Results for Experiment 1. Upper panels plot, for the peripheral (two leftmost) and central (two rightmost) conditions, T1 accuracy (left) and response times (right) as a function of stimulus-onset asynchrony (SOA) and T2 duration. T2 duration is indicated by line darkness, from darkest gray (most difficult) to lightest gray (least difficult) representing values of 133, 150, 166, and 183 ms, respectively. Lower panels plot percentage correct T2|T1 responses in the peripheral (left) and central (right) T1 conditions as a function of SOA and T2 duration

Figure 2 (upper panels) displays group mean T1 accuracy as a function of T2 position (peripheral or central), SOA, and T2 duration. No effect reached statistical significance. There was, however, a marginal effect of T2 position, *F*(1, 22) = 2.92, *p* = 0.102, ${\eta}_p^2$ = 0.117, with slightly (*M* = 4.4%) higher T1 accuracy in the central T2 presentation condition. There was also a marginal T2 Position × T2 Duration interaction, *F*(3, 66) = 2.13, *p* = .103, ${\eta}_p^2$ = 0.089. All other *F*s < 1.06 (*p*s > .20).

##### T1 reaction times

Figure 2 (upper panels) also displays group mean RT1 as a function of T2 position (peripheral or central), SOA, and T2 duration. A significant main effect of T2 position was observed, *F*(1, 22) = 9.45, *p* = .006, ${\eta}_p^2$ = 0.301, with longer RT1 in the peripheral (*M* = 883 ms) versus central (*M* = 692 ms) T2 presentation condition. A main effect of SOA was also observed, *F*(1.15, 22.32) = 4.51, *p* = .039, ${\eta}_p^2$ = 0.170, indicating that RT1 decreased as SOA was shortened. The main effect of T2 duration was marginally significant, *F*(2.07, 45.56) = 3.055, *p* = .055. No other effect reached significance, all *F*s < 1.79 (*p*s > .19).

#### T2 accuracy

Figure 2 (lower panels) displays group mean percent T2|T1 correct responses as a function of T2 position, SOA, and T2 duration. As expected, a main effect of T2 position was observed, *F*(1, 22) = 7.93, *p* = 0.01, ${\eta}_p^2$ = 0.265, with reduced overall T2|T1 accuracy in the peripheral (*M* = 65%) versus central (*M* = 81%) presentation condition. A main effect of SOA was also observed, *F*(2, 44) = 19.827, *p* < .001, ${\eta}_p^2$ = 0.471, reflecting a decrease in T2|T1 accuracy as SOA was shortened. And finally, a main effect of T2 duration was observed, *F*(1.91, 42.04) = 46.065, *p* < .001, ${\eta}_p^2$ = 0.677, with decreasing T2|T1 accuracy as T2 duration was shortened, and masking duration was lengthened.

In addition, there was a significant T2 Position × SOA interaction, *F*(2, 44) = 3.485, *p* = .043, ${\eta}_p^2$ = 0.137, as the SOA effect was larger in the central T2 presentation condition (*M* = 12%), compared with the peripheral T2 presentation condition (*M* = 4.7%). This raises the question of whether the main effect of SOA is entirely driven by a central vision effect. To ascertain this was not the case, and that there was also a PRP effect in the peripheral condition, we ran a separate 3 (SOA) × 4 (T2 presentation duration) repeated-measures ANOVA on peripheral T2|T1 accuracies. Fortunately, there was a significant main effect of SOA, though its effect size was more modest, *F*(2, 22) = 3.67, *p* = .042, ${\eta}_p^2$ = 0.25. No other effect reached significance, all *F*s < 1.8 (*p*s < .13).

### Discussion

As expected, T2|T1 accuracy decreased as SOA was shortened. Indeed, there was significant PRP interference on T2|T1 accuracy in the peripheral condition, and though its size was more modest compared with the central condition PRP effect, it was nonetheless in line with the 5% reduction observed in a methodologically comparable study (Brisson & Jolicoeur, 2007c). Surprisingly however, not only did removing the spatial shift component from the task not reduce PRP interference on T2|T1 accuracy, but it appeared to increase interference (this result will be further explored in the General Discussion). This is a strong indication that spatial attention shift was not the main cause of PRP interference observed by Brisson and Jolicoeur (2007b).

## Experiment 2: Sensory modality

Having eliminated the confound of visual-spatial attention shifts, Experiment 2 built upon the design of Experiment 1 to remove a second potential confound—namely, sensory modality switching (all the while still controlling for visual-spatial attention). To this end, Experiment 2 was identical to the central condition of Experiment 1, except that a visual T1 was substituted to the auditory T1 in half of the trials. The visual T1 in question was one of four rectangles, each with a different width.

Were the SOA effect on T2 accuracy to remain when T1 is presented in the same sensory modality as T2 (i.e., visual T1, visual T2), then this would indicate that modality switching (i.e., from auditory to visual modality) in a PRP dual task paradigm is not by itself sufficient to explain early perceptual effects observed by Brisson and Jolicoeur (2007a, b). It would also support a commonly held assumption that presenting targets in different modalities does not hinder—and may even alleviate to a certain extent—the PRP effect (Pashler, 1994).

### Method

#### Participants

Eighteen French-speaking volunteers participated in this experiment for financial compensation. Four participants were excluded (see Experiment 1, Analyses). Of the 14 remaining participants (eight women; ages 20–27 years, mean age = 22.43 years), seven were returning participants from Experiment 1. All participants were neurologically intact and reported having normal or corrected-to-normal visual acuity and color vision, as well as normal audition. This experiment received approval from the UQTR-REC.

#### Stimuli and apparatus

**Fig. 3:**
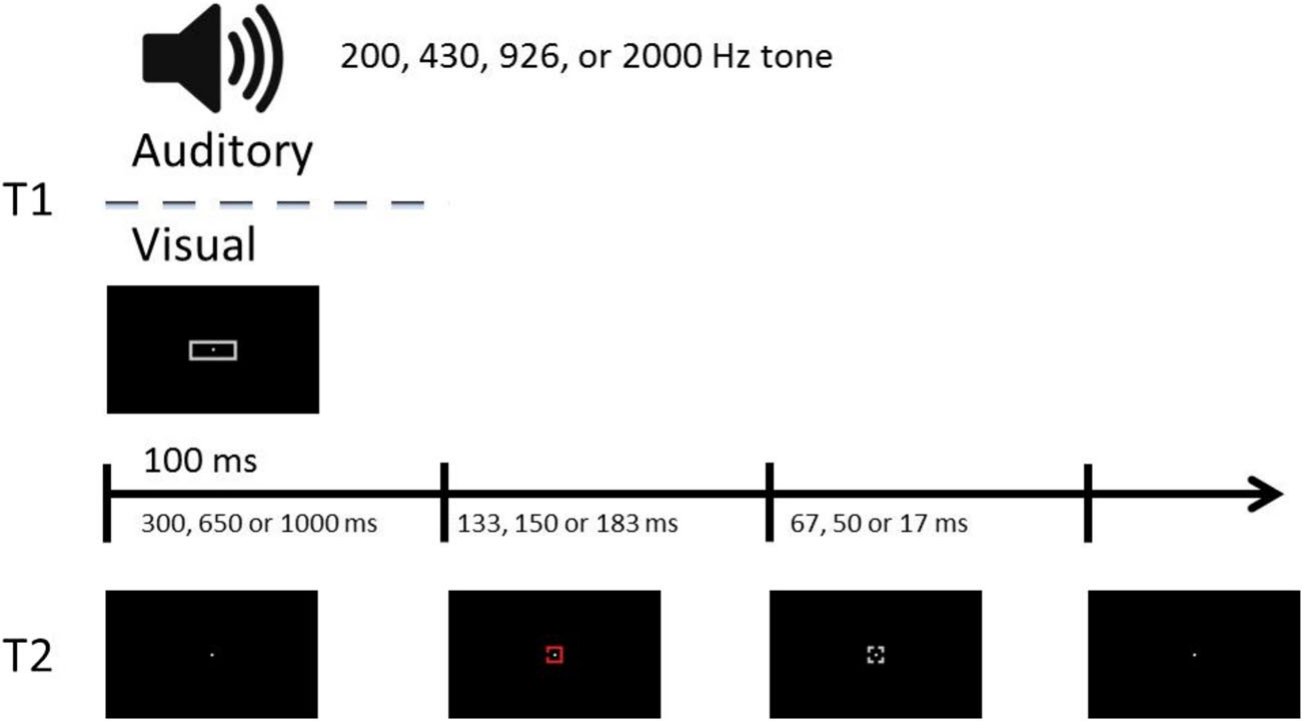
Illustration of a trial sequence from Experiment 2. T1 was presented 100 ms. It consisted of either an auditory tone or a gray rectangle to be identified. After either a 300, 650, or 1,000 ms stimulus-onset asynchrony (SOA), T2, a colored square with a gap in one of its sides, was presented at fixation until a mask (gray square with four gaps) replaced it 133, 150, or 183 ms later, and for 67, 50, or 17 ms. Participants were asked to localize the gap in T2 once they had given their response to T1

Stimuli and apparatus were identical to the central condition of Experiment 1, except for the following. As illustrated in Fig. 3, in half the trials, T1 was the same auditory tone as presented in Experiment 1. In the other half of trials, T1 was a visual rectangle presented for the same duration as the auditory T1 in Experiment 1 (100 ms). It subtended a height of 0.6°, and a width of 1.6°, 1.4°, 1.2°, or 1°. Possible T2 presentation durations were 133, 150, and 183 ms (accompanied by a 67, 50, or 17 ms mask, respectively).

#### Design and procedure

A within-subjects design was employed with three factors: T1 sensory modality (auditory or visual), SOA (300, 650, or 1,000 ms), and T2 duration (133, 150, or 183 ms). T1 modality was counterbalanced between blocks, and administration order counterbalanced between subjects. SOA and T2 duration were randomly intermixed across trials within each block.

Though the shortest stimulus onset asynchrony (300 ms) in this study can seem long compared with other PRP studies (e.g., 100 or 200 ms in Lien et al., 2011), this parameter was selected to avoid any possibility of T1 acting as a visual mask to T2 in the within-sensory modality condition (see also General Discussion, Limits). In accordance, a more difficult four-alternative Task 1 was favored over typical two-alternative iterations in order to prolong central processing of T1 and ensure robust PRP effects at 300 ms SOA (Lien et al., 2011).

As in Experiment 1, the experiment began with the presentation of written instructions on-screen. For participants (*N* = 7) who started with the auditory T1, familiarization, practice, and experiment were carried out as in Experiment 1, with the same number of trials. After a break, they completed the same familiarization, practice, and experiment sequence; however, in this run, a visual T1 replaced the auditory T1, and participants were instead required to report rectangle width. For the other half of participants, whom started with the visual T1, the order was reversed. In all, participants completed 768 experimental trials (384 each for the auditory and visual T1 target conditions).

Response inputs for the auditory T1, and for T2, were identical to Experiment 1. For visual T1 targets, participants had to report rectangle width. Responses to the length of the rectangle (visual T1) were given using the left hand, pressing on the appropriate keyboard keys: “A” (pinky; widest), “S” (ring; wide), “D” (middle; narrow), and “F” (index; narrowest).

#### Analyses

Experiment 2 mean T1 accuracy, RT1, and T2|T1 accuracy were each analyzed with repeated measures ANOVA with three within-subject factors: T1 modality (auditory, and visual), SOA (300, 650, and 1,000 ms), and T2 duration (133, 150, and 183 ms).

### Results

#### T1 performance

##### T1 accuracy

**Fig. 4:**
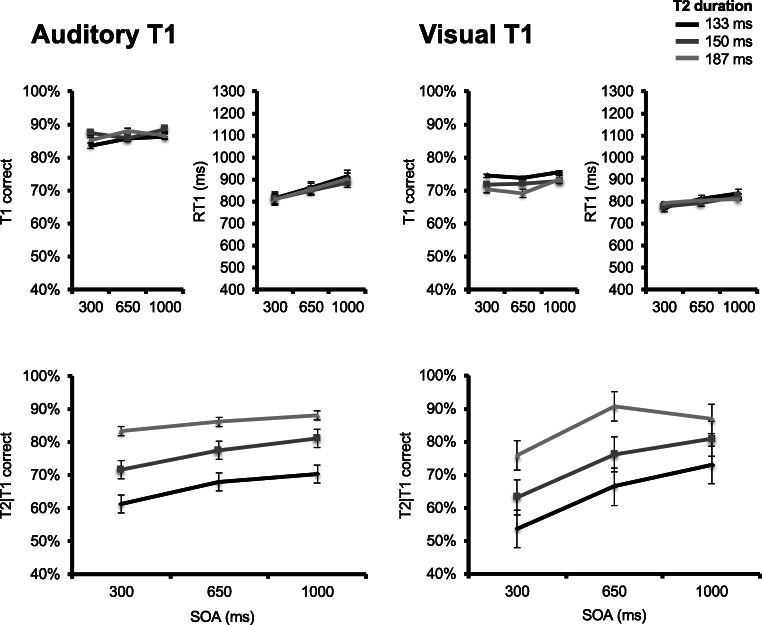
Results for Experiment 2. Upper panels plot, for the auditory (two leftmost) and visual (two rightmost) conditions, T1 accuracy (left) and response times (right) as a function of stimulus-onset asynchrony (SOA) and T2 duration. T2 duration is indicated by line darkness, from darkest gray (most difficult) to lightest gray (least difficult) representing values of 133, 150, and 183 ms, respectively. Lower panels plot percentage correct T2|T1 responses in the auditory (left) and visual (right) T1 conditions as a function of SOA and T2 duration

Figure 4 (upper panels) displays group mean T1 accuracy as a function of T1 modality, SOA, and T2 duration. A main effect of T1 modality was observed, *F*(1, 13) = 38.125, *p* < .001, ${\eta}_p^2$ = 0.746, with lower T1 accuracy in the visual (*M* = 75%) versus auditory (*M* = 88%) T1 condition. A significant T1 Modality × T2 Duration interaction effect was also observed, *F*(2, 26) = 6.345, *p* = .001, ${\eta}_p^2$ = 0.328. No other effect reached significance, all *F*s < 1.07 (*p*s > .35).

##### T1 reaction times

Figure 4 (upper panels) also displays group mean RT1 as a function of T1 modality, SOA, and T2 duration. There was a marginal SOA effect, *F*(2, 26) = 3.24, *p* = .056, ${\eta}_p^2$ = 0.2, resulting in shorter RT1 at shorter SOAs. A significant T1 Modality × SOA interaction was also observed, *F*(1.35, 17.57) = 6.146, *p* = .017, ${\eta}_p^2$ = 0.321, as the SOA effect on RT1 was larger in the auditory versus visual T1 condition. No other effect reached significance, all *F*s < 1.53 (*p*s > .23).

#### T2 accuracy

Figure 4 (lower panels) displays group mean percentage of correct T2|T1 responses, given correct T1 responses as a function of T1 modality, SOA, and T2 duration. A main effect of SOA was observed, *F*(1.3, 16.89) = 17.03, *p* < .001, ${\eta}_p^2$ = 0.567, reflecting T2|T1 accuracy decrease as SOA was shortened. A main effect of T2 duration was also observed, *F*(1.18, 15.33) = 27.979, *p* < .001, ${\eta}_p^2$ = 0.683, with lower T2|T1 accuracy as T2 duration was shortened, and masking was lengthened. There also was a significant SOA × T2 Duration interaction, *F*(2.61, 33.87) = 5.208, *p* = .006, ${\eta}_p^2$ = 0.286, indicating a more modest effect of SOA at longer T2 durations. Finally, there was a marginal T1 Modality × SOA interaction, *F*(1.28, 16.59) = 3.786, *p* = 0.061, ${\eta}_p^2$ = 0.226, indicating the SOA effect tended to be larger in the visual T1 condition. No other effect reached significance, all *F*s < 1.51 (*p*s > .22).

### Discussion

The main goal of Experiment 2 was to verify if modality switching costs could account for the supposedly early perceptual PRP effects observed by Brisson and Jolicoeur (2007b). Thus, T1 sensory modality was manipulated in a block design, from auditory (i.e., modality switch) to visual (i.e., no modality switch). The fact that a PRP effect on T2|T1 accuracy was observed in both T1 sensory modalities indicates that sensory modality switching alone cannot account for previous findings. In fact, cross-modality processing of T1–T2 was even marginally beneficial, compared with within-modality processing. We further discuss this in the General Discussion. Nonetheless, the fact remains: There was substantial PRP interference in the absence of sensory modality change from T1 to T2 processing.

## Experiment 3: Task switching

As Experiment 2 built upon Experiment 1, Experiment 3 built upon the design of Experiment 2 to test the effect of a third possible confound of early PRP interference—namely, task switching. Experiment 3 was identical to Experiment 2, except that a condition with a visual T1 similar to T2 was substituted to the auditory T1 condition; thus, in this condition, the visual T1 also involved localizing a gap in a square. If a PRP effect on T2 accuracy is still present when no task switching is involved, then we may conclude that task switching cannot fully account for early PRP effects reported by Brisson and Jolicoeur (2007a, b).

### Method

#### Participants

Seventeen French-speaking volunteers participated in this experiment for financial compensation. Three participants were excluded (see Analyses, below). Of the 14 remaining participants (10 women; ages 19–26 years, mean age = 22.43 years), nine were returning participants from either of the preceding experiments. The experiment was approved by the UQTR-REC.

#### Stimuli and apparatus

**Fig. 5:**
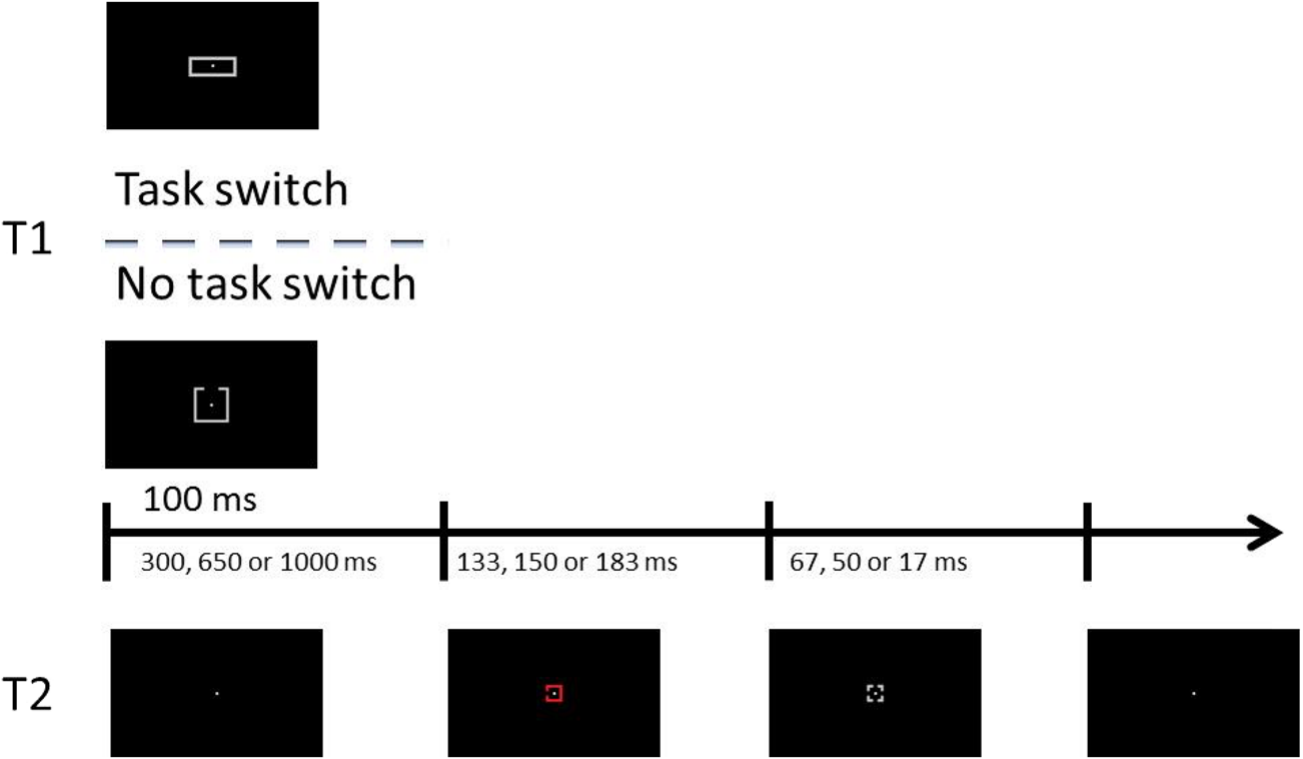
Illustration of a trial sequence from Experiment 3. T1 was presented 100 ms. It consisted of either a gray rectangle to identify, or a gray square with a gap in one of its sides, to be located. After either a 300, 650, or 1,000 ms stimulus-onset asynchrony (SOA), T2, a color square with a gap in one of its sides, was presented at fixation until a mask (gray square with four gaps), replaced it 133, 150, or 183 ms later, and for 67, 50, or 17 ms. Participants were asked to localize the gap once they had given their response to T1

All stimuli and apparatus were identical to Experiment 2, with the following exceptions. As illustrated in Fig. 5, T1 was always presented in the visual modality. In half of the trials, possible T1 targets were rectangles of varying widths, as in Experiment 2. In the other half of trials, possible T1 targets were four gray squares (2° × 2°), each with a 0.67° gap in one of its sides.

#### Design and procedure

A within-subjects design was employed with three factors: T1 task type (rectangle width [task switch], and square gap [no task switch]), SOA (300, 650, and 1,000 ms), and T2 duration (133, 150, and 183 ms). T1 task type was counterbalanced between blocks (with administration order counterbalanced between subjects), whereas the two other factors were randomly intermixed across trials within each block.

The procedure was identical to Experiment 2, except that for the square gap T1 (no task switching), participants performed the same task as they did in T2 (i.e., report the location of the gap). Gap localization responses were provided with the left hand using the following keys: “A” (pinky; left), “S” (ring; bottom), “D” (middle; top) and “F” (index; right).

#### Analyses

Experiment 3 mean T1 accuracy, RT1, and T2|T1 accuracy were each analyzed with a repeated-measures ANOVA, with three within-subjects factors: T1 task type (rectangle width [task switch], and square gap [no task switch]), SOA (300, 650, and 1,000 ms), and T2 duration (133, 150, and 183 ms).

### Results

#### T1 performance

##### T1 report accuracy

**Fig. 6:**
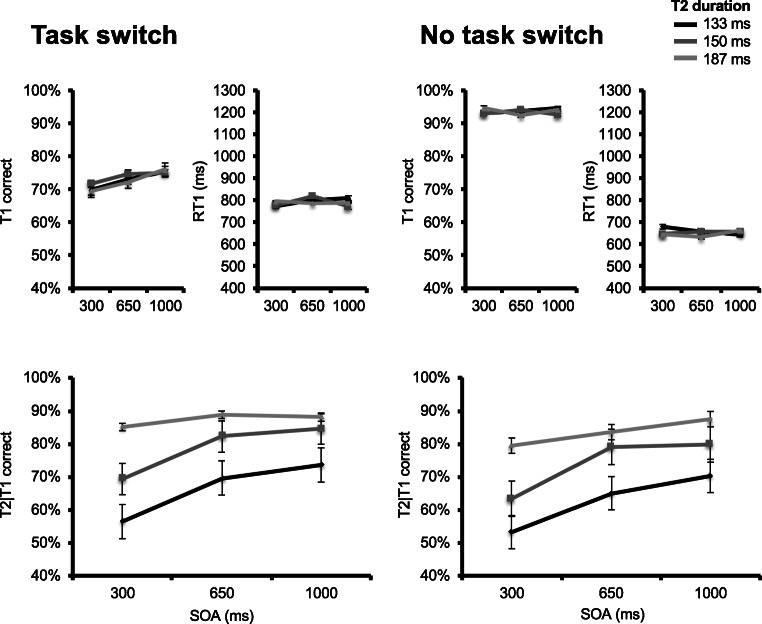
Results for Experiment 3. Upper panels plot, for the rectangle length (two leftmost) and square gap (two rightmost) conditions, T1 accuracy (left) and response times (right) as a function of stimulus-onset asynchrony (SOA) and T2 duration. T2 duration is indicated by line darkness, from darkest gray (most difficult) to lightest gray (least difficult) representing values of 133, 150, and 183 ms. Lower panels plot percentage correct T2|T1 responses in the task switching (left) and no task switching (right) condition as a function of SOA and T2 duration

Figure 6 (upper panels) displays group mean T1 accuracy as a function of T1 task type, SOA, and T2 duration. A main effect of T1 task type was observed, *F*(1, 13) = 54.139, *p* < .001, ${\eta}_p^2$ = 0.806, with lower T1 accuracy in the rectangle width (*M* = 74%) versus square gap (*M* = 94%) task. A main effect of SOA was also observed, *F*(2, 26) = 4.854, *p* = .016, ${\eta}_p^2$ = 0.272, with slightly lower T1 accuracy in the shortest 300 ms (*M* = 82%) SOA condition versus the 650 ms (*M* = 84%) and 1,000 ms (*M* = 84%) conditions. No other effect reached significance, all *F*s < 1.49 (*p*s > .24).

##### T1 reaction times

Figure 6 (upper panels) also displays group mean response times to T1 as a function of T1 task type, SOA, and T2 duration. A significant main effect of T1 task type was observed, *F*(1, 13) = 51.535, *p* < .001, ${\eta}_p^2$ = 0.799, with slower response times to T1 in the rectangle width (*M* = 737 ms) versus square gap (*M* = 622 ms) task. No other effect reached significance, all *F*s < 1.75 (*p*s > .18).

#### T2 accuracy

Finally, Fig. 6 (lower panels) displays group mean correct T2|T1 correct responses as a function of T1 task type, SOA, and T2 duration. A main effect of T1 task type was observed, *F*(1, 13) = 13.169, *p* = .003, ${\eta}_p^2$ = 0.503, with higher T2|T1 accuracy when T1 involved task switching (i.e., rectangle width, *M* = 82%) versus when no task switching was involved (i.e., square gap, *M* = 77%). As in the two previous experiments, a main effect of SOA was observed, *F*(1.22, 15.85) = 18.433, *p* < .001, ${\eta}_p^2$ = 0.586, indicating a decrease in accuracy as SOA was shortened. A main effect of T2 duration was observed as well, *F*(1.1, 14.35) = 25.21, *p* < .001, ${\eta}_p^2$ = 0.66, indicating a decrease in T2|T1 accuracy as T2 duration was decreased. Finally, there was a significant SOA × T2 Duration interaction, *F*(2.62, 34.05) = 8.493, *p* < .001, ${\eta}_p^2$ = 0.395, with a smaller T2 duration effect as SOA increased and task overlap decreased. No other effect reached significance, all *F*s < 1 (*p*s > .47). Importantly, this is also true of the SOA × T1 Task Type interaction *F*(2, 26) = 0.472, *p* = .629, ${\eta}_p^2$ = 0.035, which implicates task switching was not the main source of interference on T2 processing.

### Discussion

It has been postulated that task switching can interfere with processing at a relatively early stage (Vachon, Jolicoeur, 2011; Vachon, Tremblay, & Jones, 2007). The main goal of Experiment 3 was to verify if this could explain early PRP effects. Thus, task type was manipulated in a block-design, from a dual task with transition from T1 to T2 processing requiring task switching (report rectangle width of T1), to a dual task with transition from T1 to T2 processing not requiring task switching (localize a gap on the T1 square). The observed PRP effect on T2|T1 accuracy was similar across task switching and no task switching conditions in spite of T1 also being easier in the latter case, as indicated by the absence of an interaction between SOA and T1 task type on T2 accuracy. Thus, though task switching from T1 to T2 (Vachon Jolicoeur, 2011; Vachon et al., 2007) and increased T1 processing difficulty (Brisson & Jolicoeur, 2007a, b, c; Lien et al., 2011) might cause central interference affecting T2 processing, we find no evidence that these factors were the main source of early PRP interference reported by Brisson and Jolicoeur (2007a, b).

## Experiment 4: Response modality

As was the case for the previous experiments, Experiment 4 built upon the design of Experiment 3 to test the effect of a fourth and final possible cause for the early PRP interference observed by Brisson and Jolicoeur (2007c): changes in response modality from T1 to T2. Specifically, in Experiments 1–3, participants responded to T1 with their left hand, and switched to their right hand to give their response to T2. Thus, in this final experiment, we designed a procedure whereby participants responded to both T1 and T2 using the same hand to verify whether hand switching could account for early PRP interference. In the present experiment, T1 and T2 consisted of a gap localization. However, in one half of trials, participants responded with their left hand to T1, and with their right hand to T2 (hand switch condition); and in the other half, they responded with their right hand to both T1 and T2 (no hand switch condition).

### Method

#### Participants

Sixteen French-speaking volunteers participated in this experiment for financial compensation. Four participants were excluded (see Experiment 1, Analyses). Among the 12 remaining participants (seven women; ages 21–26 years, mean age = 22.5 years), nine had participated in at least one of the three prior experiments. All participants were neurologically intact and reported having normal or corrected-to-normal visual acuity and color vision, as well as normal audition. The experiment was approved by the UQTR-REC.

#### Stimuli and apparatus

**Fig. 7:**
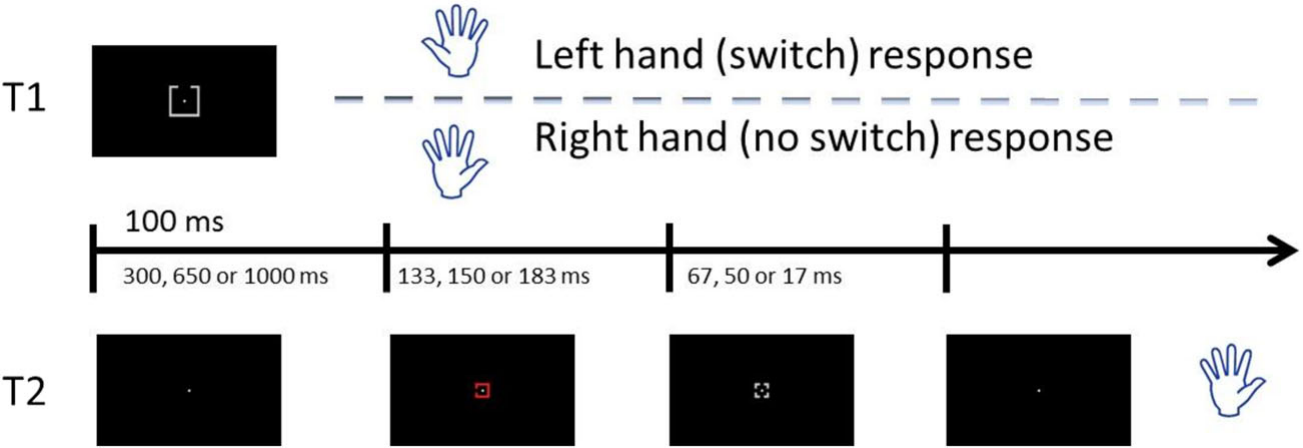
Illustration of a trial sequence from Experiment 4. T1 was presented 100 ms. It consisted of a gray square with a gap in one of its sides, which participants had to localize. They did so either with the left hand (switch condition) or the right hand (no switch condition). After either a 300, 650 or 1,000 ms stimulus-onset asynchrony (SOA), T2, a colored square with a gap in one of its sides, was presented at fixation until a mask (gray square with four gaps), replaced it 133, 150, or 183 ms later, and for 67, 50, or 17 ms. Participants were asked to localize the gap with their right hand once they had given their response to T1

All stimuli and apparatus were identical to Experiment 3, except that on every trial, T1 was a gray square (2° × 2°) with a 0.67° gap on one of its sides, presented at fixation (see Figure 7).

#### Design and procedure

A within-subjects design was employed with three factors: T1 response hand (left, and right), SOA (300 ms, 650 ms, and 1,000 ms), and T2 duration (133, 150, and 183 ms). T1 response hand was counterbalanced between blocks of trials (with this order counterbalanced between subjects), whereas the two other factors were randomized across trials within each block.

T2 responses were always given using the right hand (same keys as for T2 in Experiments 1–3; i.e., “J” [index; left], “K” [middle; bottom], “L” [ring; top], and “;” [pinky; right]). In one half of the trials, participants responded to T1 with their left hand (same keys as T1 in Experiment 3; i.e., “A” [pinky; left], “S” [ring; bottom], “D” [middle; top], and “F” [index; right]). In the other half of trials, participants responded to both T1 and T2 using their right hand (same keys as for T2).

#### Analyses

Experiment 4 mean T1 accuracy, RT1, and T2|T1 accuracy were each analyzed with a repeated-measures ANOVA, with three within-subjects factors: T1 response hand (left [hand switch], and right [no hand switch]), SOA (300, 650, and 1,000 ms), and T2 duration (133, 150, and 183 ms).

### Results

#### T1 performance

##### T1 report accuracy

**Fig. 8:**
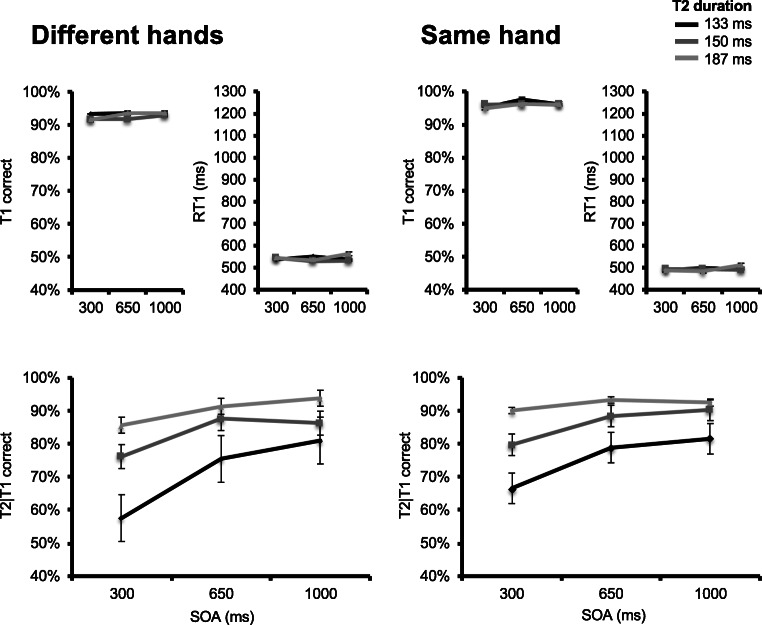
Results for Experiment 4. Upper panels plot, for both hand switching (two leftmost) and no hand switching (two rightmost) conditions, T1 accuracy (left) and response times (right) as a function of stimulus-onset asynchrony (SOA) and T2 duration. T2 duration is indicated by line darkness, from darkest gray (most difficult) to lightest gray (least difficult) representing values of 133, 150, and 183 ms. Lower panels plot T2|T1 correct responses in the hand switching (left) and no hand switching (right) conditions as a function of SOA and T2 duration

Figure 8 (upper panels) displays group mean T1 accuracy as a function of T1 response hand, SOA, and T2 duration. A main effect of T1 response hand was observed, *F*(1, 11) = 8.627, *p* = .014, ${\eta}_p^2$ = 0.44, with slightly lower T1 accuracy in the left (*M* = 95%) versus right (*M* = 97%) hand condition. No other effect reached significance, all *F*s < 2.17 (*p*s > .15).

##### T1 reaction times

Figure 8 (upper panels) also displays group mean response times to T1 as a function of T1 response hand, SOA, and T2 duration. A main effect of T1 response hand was observed, *F*(1, 11) = 49.239, *p* < .001, ${\eta}_p^2$ = 0.817, with slower response times to T1 in the left (*M* = 511 ms) versus right (*M* = 455 ms) hand condition. No other effect reached significance, all *F*s < 1.41 (*p*s > .26).

#### T2 accuracy

Figure 8 (lower panels) displays group mean correct T2|T1 correct responses as a function of T1 response hand, SOA, and T2 duration. The main effect of T1 response hand was marginally significant, *F*(1, 11) = 3.519, *p* = .087, ${\eta}_p^2$ = 0.242, as T2|T1 performance was slightly better when response hand was the same for T1 and T2. A main effect of SOA was observed, *F*(1.22, 13.39) = 32.202, *p* < .001, ${\eta}_p^2$ = .745, indicating better T2|T1 accuracy at longer SOA values. A significant main effect of T2 duration was observed as well, *F*(1.23, 13.57) = 27.676, *p* < .001 ${\eta}_p^2$ = 0.716, indicating that T2|T1 accuracy increased as T2 duration was lengthened, and masking shortened.

A significant SOA × T2 Duration interaction effect was observed, *F*(2.07, 22.73) = 9.422, *p* = .001, ${\eta}_p^2$ = 0.461, indicating that the effect of T2 stimulus duration on T2|T1 accuracy was smaller at longer SOA values. There also was a marginal T1 Response Hand × SOA interaction, *F*(2, 22) = 2.744, *p* = .099, indicating the SOA effect on T2|T1 accuracy was slightly reduced when no response hand switching was required (*M* = 10.6%), compared with when hand switching was required (*M* = 15.6%). We therefore conducted a secondary repeated-measures ANOVA, with SOA and T2 duration as within-subjects factors to ensure that the SOA effect was not solely driven by hand switching. This analysis revealed a significant SOA effect when no hand switching was required *F*(2, 22) = 16.43, *p* < .001, ${\eta}_p^2$ = 0.599. No other effect reached significance, all *F*s < 2.59 (*p*s > .12).

### Discussion

The main goal of Experiment 4 was to exclude T1–T2 hand switching as a possible locus of PRP interference in the results reported by Brisson and Jolicoeur (2007a, b). To this end, we introduced a condition in which there was no hand/key switching between tasks, and T1 and T2 responses were thus given using the same hand and keys. This marginally reduced the PRP effect on T2|T1 accuracy as compared with when a hand/key switching was required from T1 to T2, though a strong PRP effect remained even when no hand switching was required. That is, there remained a significant interaction between SOA and T2 duration, such that the PRP effect on T2 accuracy was amplified as a function of task difficulty even when no hand switching was required. Hence, though response hand/key switching might cause central interference, it does not on its own explain early interference effects that were observed on T2 accuracy.

## General discussion

We aimed to explore four possible nonperceptual loci of psychological refractory period (PRP) interference. To this end, we systematically and iteratively tested visual-spatial attention, sensory modality switching, task switching, and response modality switching in a series of behavioral experiments. Despite controlling all these aspects, interference remained manifest.

### Evidence for early perceptual interference

In addition to later cognitive processing stages coinciding with more classical accounts of PRP, Brisson and Jolicoeur (2007b) also found electrophysiological evidence of early perceptual interference affecting perceptual encoding (as indexed by reduced P1 and N1 amplitudes), visual-spatial attention (as index by a reduced N2pc component) and visual short-term memory (vSTM) consolidation (as indexed by a delayed SPCN component; see also, for analogous effects, Brisson & Jolicoeur, 2007a, c; Brisson, Leblanc, & Jolicoeur, 2009). Using a more classical PRP paradigm (compared with Brisson & Jolicoeur, 2007b), we removed four possible confounding loci of interference. In spite of this, our study still found clear evidence of PRP interference, such that T2 processing—as reflected by response accuracy—was systematically impaired at shorter versus longer SOA.

First, were shifts in visual-spatial attention the main locus of the PRP effect, we should have observed a reduced or even abolished SOA effect when T2 were presented centrally, compared with when they were presented peripherally. Yet the reverse pattern was obtained: PRP appeared amplified in central vision. The inescapable conclusion of this surprising finding is that visual perception in the fovea is also susceptible to PRP interference. One might even hypothesize that foveal vision is more susceptible to central interference, compared with peripheral vision, as the drop in T2 accuracy in the former more than doubled what was seen in the latter. Indeed, the PRP-induced 4.7% reduction in T2|T1 accuracy in the peripheral viewing condition is similar to the 5% reduction observed in a comparable study (Brisson & Jolicoeur, 2007c), suggesting that the present PRP parameter estimate is not an oddity. At the same time, overall accuracy in the peripheral condition of the present study was about 15% lower compared with the study of Brisson and Jolicoeur (2007c), but also compared with our central viewing condition. Thus, intersubject variability likely accounts for at least part of the discrepancy between peripheral and central targets—as this was a between-subjects factor.

However, we cannot rule with certainty on the possibility that central interference is greater in foveal versus peripheral vision from the current data alone, since condition accuracies are not directly comparable. For instance, stimulus size of peripheral targets (3.5° of eccentricity) was not adjusted to compensate for visual acuity loss in peripheral vision. Additionally, targets in the peripheral condition, but not the central condition, were surrounded by distractors, increasing discrimination confusability in this condition. Together, this may have created dissociate performance ceilings across peripheral and central viewing conditions—though how exactly this could have affected PRP magnitude is unclear. Future work should more carefully control these parameters to make a more direct comparison between performances in central and peripheral vision more straightforward. It should also rely on a within-subjects design, rather than a mixed design. One thing is clear, however: Taking spatial attention out of the picture does not eliminate PRP effects, indicating that the locus of interference lies elsewhere in the T2 processing sequence.

Second, were shifts in sensory modality the locus of PRP interference, we should have observed an elimination (or reduction) of the PRP effect once this confound was removed in Experiment 2. Contrary to this prediction however, there was no reduction of the PRP effect when sensory modality switch from T1 to T2 processing was required—in fact, this led to a marginally larger SOA effect. Given that T2 performance here can be compared on common grounds (contrary to Experiment 1), the slight increase in PRP interference when no sensory modality switching was required hints at within-modality interference at short SOAs that could arise as a consequence of processing T1 and T2 within the same sensory modality (see Pashler, 1994; see Limits, below, for further discussion of this issue). In any case however, this definitely excludes sensory modality switching from T1 processing to T2 processing as the main locus of PRP interference.

Third, were task switching the main locus of PRP interference, then removing the need for task switching should have reduced or eliminated PRP interference in Experiment 3. Once more, however, PRP effects remained substantial. In fact, PRP interference was virtually unchanged, whether task switching occurred from T1 to T2 or not. There remains the possibility that PRP effects in the task switch and no task switch conditions were caused by within-sensory modality interference. We do not believe this to be the case, however, and we argue below (see Limits) that within-sensory modality interference is in fact insufficient to entirely account for the observed PRP effect.

Fourth and finally, were response-modality switching the main locus of PRP interference, we should have observed a substantial reduction or elimination of PRP effects when T2 response modality was the same in Experiment 4. Despite a marginal decrease of PRP interference when the same response modality was used, there nonetheless remained strong interference. Thus, though response modality switching might have been a locus of PRP interference, it clearly was not the sole locus of interference in Experiment 4. As above, there remains the possibility that residual PRP interference in the no hand switch condition was caused by within-sensory modality interference (see Limits for an explanation as for why we do not believe this to be the case).

Having eliminated each of these confounds—visual-spatial attention, sensory modality switching, task switching, and response modality switching—what is left must reflect early perceptual interference afflicting either perceptual encoding, vSTM consolidation, or both. We already know from electrophysiological markers that these two scenarios are equally possible, and not necessarily exclusive. Indeed, Brisson and Jolicoeur (2007b) found simultaneous evidence of interference on visual encoding as well as a delay in vSTM consolidation. Interestingly, this was also reflected in T2 report accuracy, which though very brief (50 ms), was not masked. The implication is that, when a speed constraint on Task 1 is combined with a perceptual constraint on Task 2 (using very brief presentation time or masking), T2 information may not get fully encoded, and what does get encoded is more susceptible to decay as it awaits/undergoes consolidation in vSTM. Such perceptual processing may serve for simple detection, but it would be less dependable for more complex discrimination.

Furthermore, the fact that masking was not caused by a tailing distractor in our study means it is unlikely the drop in T2 accuracy occurred as a result of interference during short-term memory updating. Indeed, in our study, the accuracy drop at short SOA was much more modest—or even abolished—when T2 presentation/masking duration was longest/shortest, respectively. This indicates that, provided perceptual processing of T2 reaches a certain threshold (determined by task demands), it will successfully be reported, albeit in a delayed fashion—producing the classical PRP effect. A more likely explanation, then, is that our manipulation, by inducing both a speed constraint on T1 processing and a perceptual constraint on T2 processing, impeded early perceptual processing of T2, either preventing T2 from being sufficiently encoded, or causing T2 to decay as it awaited/underwent vSTM consolidation. Here, well-versed readers may notice a resemblance to attentional blink, to which we now turn our attention.

### Parallel between PRP and AB

AB and PRP effects have always constituted a strange duality, and the present study makes this all the more manifest. As mentioned in the introduction, with each iteration, our PRP task variants more and more resembled an attentional blink (AB) paradigm. AB tasks typically require participants to detect or identify two targets embedded in a rapid visual presentation of distractor items while the temporal distance (and number of distractors) between targets is manipulated. Contrary to PRP paradigms, however, this type of dual task is usually carried out without speed constraint. Classically, report accuracy of T2 declines as the temporal lag between targets is reduced. Importantly for our purpose, however, this effect is also observed in minimal AB settings—a variant in which there is no distractor string and targets are instead each followed by a single mask (e.g., Duncan, Ward, & Shapiro, 1994). As such, our tasks (at least, those presented in Experiments 2–4) can be conceptualized as PRP with an AB variant (target masking), and nearly equally as (minimal) AB with a PRP variant (speed constraint).

Although there existed a debate about whether AB was caused by T1 processing requirements (i.e., capacity-limited models; Chun & Potter, 1995; Wyble, Bowman, & Nieuwenstein, 2009) or intervening distractors (i.e., distractor-based models; Di Lollo, Kawahara, Ghorashi, & Enns, 2005; Olivers & Meeter, 2008; Raymond et al., 1992), distractor-based models have essentially been rejected (e.g., Lagroix, Spalek, Wyble, Jannati, & Di Lollo, 2012; Nieuwenstein, Potter, & Theeuwes, 2009)—which is not to say intervening distractors cause no interference whatsoever (e.g., when no task switch is required between targets; Brisson, 2015; Brisson, Spalek & Di Lollo, 2011). Capacity-based models assume that two targets cannot be consolidated in short-term memory at the same time. While T1 is being consolidated, T2 is subjected to decay (Chun & Potter, 1995), to masking (Giesbrecht & Di Lollo, 1998; Vogel & Luck, 2002) or misselection (Bourassa, Vachon, & Brisson, 2015; Nieuwenstein et al., 2009; Vul, Nieuwenstein, & Kanwisher, 2008). Interestingly, a recent electrophysiological study (Dell’Acqua et al., 2015) linked AB to both a decrease of target detection efficiency (as indicated by P3a amplitude reduction) and delayed short-term consolidation (as indicated by a delayed P3b).

In short, when the task puts constraints on consolidation and temporal selection, as in classical unspeeded AB paradigms, prominent models assume that consolidation of T1 in short-term memory is the main locus of interference, causing a delay in T2 short-term memory consolidation, but also affecting early detection/selection of the latter (see for review Dux & Marois, 2009; Martens & Wyble, 2010). On the other hand, when the task puts constraints on response selection, such as in PRP paradigms, prominent models assume that response selection of T1 is the main locus of interference, causing a delay in T2 response selection.

This illustrates how AB and PRP effects have typically been understood to reflect different processing resource limitations. However, seeing as observations are constrained by methodology, the fact that AB and PRP effects have classically been measured with two different paradigms may have contributed to their being conceptualized as distinct. Some have tried to bridge this gap between AB and PRP by crossing the methodological boundary. Pierre Jolicoeur (1999), for example, introduced PRP elements to his AB paradigm by introducing a speeded four-alternative discrimination Task 1 in an AB dual task (see also Arnell & Jolicoeur, 1999; Dell’Acqua, Jolicoeur, Pesciarelli, Job, & Palomba, 2003). Results showed that adding an online response selection constraint increased AB, leading to the central interference model—itself a more general instance of capacity-limited models—which posits that the resource-limited central stage encompasses both short-term consolidation and response selection (Jolicoeur, 1999).

Taking the reverse approach, we crossed methodological boundaries by adding consolidation constraints to T2, on top of having response selection constraints. In so doing, we showed that response selection in one task not only interferes with response selection in the other task, but also that it interferes with perceptual processing of the other task, prior to or during consolidation. That is, we revealed that central processing resource scarcity in a PRP paradigm causes an effect that is very much analogous to an attentional blink, providing further support for the central interference theory. Moreover, we found this effect to be dose dependent: Reducing perceptual/consolidation constraint by increasing T2 presentation time and decreasing masking duration led to a much more modest—or outright abolished—the drop in T2 report accuracy. Again, this is reminiscent of AB in that when T2 is unmasked, the accuracy drop is markedly reduced or abolished (e.g., Giesbrecht & Di Lollo, 1998; Vogel & Luck, 2002); which is itself reminiscent of PRP in that unless T2 is masked, report accuracy is mostly unaffected. Thus, it would seem we have come full circle.

Taking things one step further, one study even suggested that AB and PRP merely reflect intertrial fluctuations (Marti, Sigman, & Dehaene, 2012). To reach this conclusion, its authors had participants perform a dual task that consisted of a speeded two-alternative discrimination Task 1 while undergoing magnetoencephalography (MEG) recording. In so doing, they found evidence suggesting that PRP (inferred from trials in which T2 was detected, but with delay) and AB (inferred from trials in which T2 was missed altogether) emerge from a shared cortical bottleneck. Indeed, the former was characterized by delayed frontal activation, whereas the latter was characterized by failure of frontal activation. We note, however, that the study may have failed to observe earlier effects for two reasons: first, because though speeded, T1 processing demand was modest (two-alternative discrimination), and second, because T2 only required visual detection (as opposed to visual discrimination). Though our study does not allow us to go as far as to conclude AB and PRP reflect intertrial variability (see, however, Supplementary Materials for evidence of delayed responses on correct—i.e., non-“blinked”—T2 responses), we believe it further closes the gap separating the two phenomena, supporting the central interference theory while also suggesting that central processing of a first target also impacts perceptual processing of subsequent targets, in line with previous results (Dell’Acqua et al., 2015).

Admittedly, a boundary-crossing approach invariably leaves us open to the possible objection that making methodology more similar will naturally lead to overlap in measurements. To that however, we counter that a measure must also capture something beyond its idiosyncrasies if it is to reveal any fundamental aspect of cognition. In that, we think designs that cross the boundary but still retain fundamental aspects of the original paradigm are a perfectly suitable compromise.

### Limits

As already stated, one potential limit of the present study pertains to our design—namely, that each experiment was iterative, and therefore built upon the preceding one (e.g., Experiment 1 eliminated the confound of visual-spatial attention deployment; Experiment 2, in addition to visual attention, eliminated the confound of sensory modality switching). Thus, it is possible that by adopting this iterative structure we inadvertently introduced a novel confound locus of interference at some point that was not present in the original Brisson and Jolicoeur (2007a, b) task. Same sensory modality processing of T1 and T2, for instance, caused a marginal increase of PRP interference in Experiment 2, compared with cross-modal processing. This likely carried over to Experiments 3 and 4 by virtue of their iterative design and could possibly account for a modest part of the PRP effect observed in these experiments.

This was, however, necessary to test the effect of task switching (Experiment 3), as it obligatorily implies within-modality processing. Indeed, if there is sensory modality switching from T1 to T2 processing, then T1 and T2 are different tasks, and task switching is required to respond to both stimuli. Furthermore, considering that visual masking effects only occur when the trailing item is presented within 150–200 ms of the initial target (Averbach & Coriell, 1961; Spencer & Shuntich, 1970; Vogel et al., 2006), it is relatively safe to assume perceptual processing of T1 was complete by the time T2 was presented, even at the shortest SOA (i.e., 300 ms, a value selected with this specifically in mind). These, combined to the fact that within modality processing only caused a marginal increase in PRP interference in Experiment 2, lead us to argue that same sensory interference cannot account for the large PRP effects that were observed across Experiments 2–4. Thus, we hold that results from the experiments constituting this study reflect interference in early perceptual processing arising from central processing of T1, most likely on perceptual encoding or visual short-term memory encoding (Brisson & Jolicoeur, 2007a, b, c).

### Future directions

Though early perceptual processing appears to have been the main locus of interference in our study, this must not be taken to imply that shifts in visual-spatial attention, sensory modality, task, and response modality switching are not potential loci of PRP interference. In fact, while not direct evidence of multiple loci of PRP interference, our results are nonetheless generally consistent with such a possibility. Furthermore, there is ample evidence showing that competition with a concurrent task for central resources can interfere with visual-spatial attention deployment, especially when dealing with a cognitively taxing first task (Brisson & Jolicoeur, 2007a, b, c; Brisson, Leblanc, & Jolicoeur, 2009; Lien et al., 2011; see, however, Reimer et al., 2015, 2017). Future studies should therefore be aimed at better understanding the temporal dynamics of PRP interference to better characterize these interference loci, especially by means of human electrophysiology.

Finally, though our study shows early perceptual interference, it does not make it possible to determine whether this interference arises during perceptual encoding or during visual short-term memory consolidation (or both). Future work could look at factors susceptible to—and the extent to which they do—modulate this effect. For instance, it was shown using electrophysiological and behavioral data that emotional content (e.g., facial expressions of emotions) might spare some, but not all, early perceptual processing in a PRP paradigm (Allen, Lien, & Jardin, 2017; Duncan, Dugas, Brisson, Blais, & Fiset, 2019; Roberge, Duncan, Fiset, & Brisson, 2019; Shaw, Lien, Ruthruff, and Allen, 2011). In a related fashion, if perceptual encoding is susceptible to central interference, the logical next step is to look at what information gets encoded and what information does not, and again, look at factors that may influence this dynamic (e.g., salience, task demands, low-level visual structure).

### Conclusion

We set out to test the possibility that dual tasking as implemented in a psychological refractory period (PRP) paradigm might cause interference on early perceptual processing, either perceptual encoding or visual short-term memory consolidation. We did so by controlling for various other possible loci of interference—namely, reorienting visual-spatial attention, switching sensory modality between tasks, switching tasks, and switching response modality. Even after their removal, there remained a large and systematic decrease in T2 performance when T1 and T2 onset was separated by 650 ms or less. Thus, early perceptual processing can be negatively impacted by a PRP brought upon by central processing of a concurrent task, causing a decrease in T2 report accuracy—an effect very much reminiscent of the attentional blink.

More generally, the implication is that inability to allocate top-down processing resources—such as when these are already monopolized by another task or object—can lead to a failure to encode or consolidate perceptual information. It is easy to see how this can have real-world consequences, especially when quick and transient events are implied, and a rapid response is necessary. Such is the case, for example, when driving a car, as processing delays—to say nothing of failures—can have potentially life-altering consequences.

#### Author note

This research was supported by Discovery Grant No. 402614-2011 from the Natural Sciences and Engineering Research Council of Canada (NSERC) and the UQTR Research Chairs Program awarded to B.B. The authors thank Isabelle Fafard and Marie-Ève Bourassa for helping with data collection.

#### Open practices statement

The data sets for the current study are available from the corresponding author upon reasonable request. Experiments were not part of a preregistered study.

## Electronic supplementary material

ESM 1 (DOCX 356 kb)

## Footnotes

1 We also did the same analyses without applying exclusion criteria, and results were largely the same (see Supplementary Materials for detail).
