# Supplementary material for: Dual task interference on early perceptual processing
Source: Atten Percept Psychophys. 2020 Oct 20;83(4):1777–95. doi: 10.3758/s13414-020-02158-0 (PMC8084822; doi:10.3758/s13414-020-02158-0)
Supplement: Supplementary file 1 — (DOCX 356 kb) [file 13414_2020_2158_MOESM1_ESM.docx]

**Supplemental materials**

In the present document, we first present analyses on T2|T1 accuracy without applying exclusion criteria, to ensure the inflated rejection rate did not alter our conclusions’ accuracy. Secondly, we present analyses of T2 reaction times in order to demonstrate that speed-accuracy tradeoff could not account for results presented in the associated article.

**Analyses on T2|T1 correct accuracy, without exclusion criteria** We ran every analysis presented in the Results section of the main text without applying exclusion criteria. All results were largely the same, except for some minor differences that emerged—essentially where borderline/significant (i.e., in the range 0.1 > p > 0.01) effects were concerned. It is therefore sound to conclude that the exclusion criteria had no impact on the pattern of results and conclusions presented in the article.

In Exp. 1, the marginal SOA X Viewing condition (peripheral vs. central) interaction became nonsignificant, F(2,60) = 1.679, p = 0.195, $\eta_{p}^{2}$ = 0.053. Thus, the effect of SOA on T2 accuracy was not statistically different across peripheral and central T2 viewing positions.

In Exp. 2, the marginal SOA X T1 modality interaction became significant, F(1.27, 21.66) = 5.286, p = 0.025, $\eta_{p}^{2}$ = 0.237, reflecting a 9% larger SOA effect when T1 was visual (16.6%) vs. auditory (7.6%). This is similar in absolute size to what was obtained with our final sample (main text). The significant SOA X T2 duration interaction became marginal, F(2.23 54.89) = 2.092, p = 0.107, $\eta_{p}^{2}$ = 0.11. Thus, the effect of SOA on T2 accuracy was marginally different across T2 presentation durations, with a slight increase in SOA effect as T2 presentation duration became shorter (and masking became longer).

In Exp. 3, the significant T1 task type effect became nonsignificant, F(1, 16) = 0.806, p = 0.382, $\eta_{p}^{2}$ = 0.048. However, this was entirely due to one—initially removed—subject who had extremely low T2 accuracy when T1 required task switching (28%), vs. no task switching (61%). For the rest of the group, performance was ≈ 5% higher in the task switch vs. no task switch condition, just as with our final sample (main text).

No other difference in results emerged.

**Analysis of Task 2 response times**

***Experiment 1.*** Main effect of target position, F(1, 22) = 9.4, p = 0.006, eta = 0.299, with Task 2 response times (RT2) 279 ms slower in the peripheral vs. central condition. Main effect of SOA, F(1.49, 32.81) = 94.899, p < 0.001, eta = 0.812, with RT2 241 ms slower at shortest vs. longest SOA. Main effect of T2 duration, F(3, 66) = 3.997, p = 0.011, eta = 0.154, with RT2 70 ms slower at shortest vs. longest T2 duration (and longest vs. shortest masking, respectively). No other significant effect, all *F*s < 1.1 (*p*s > 0.33).


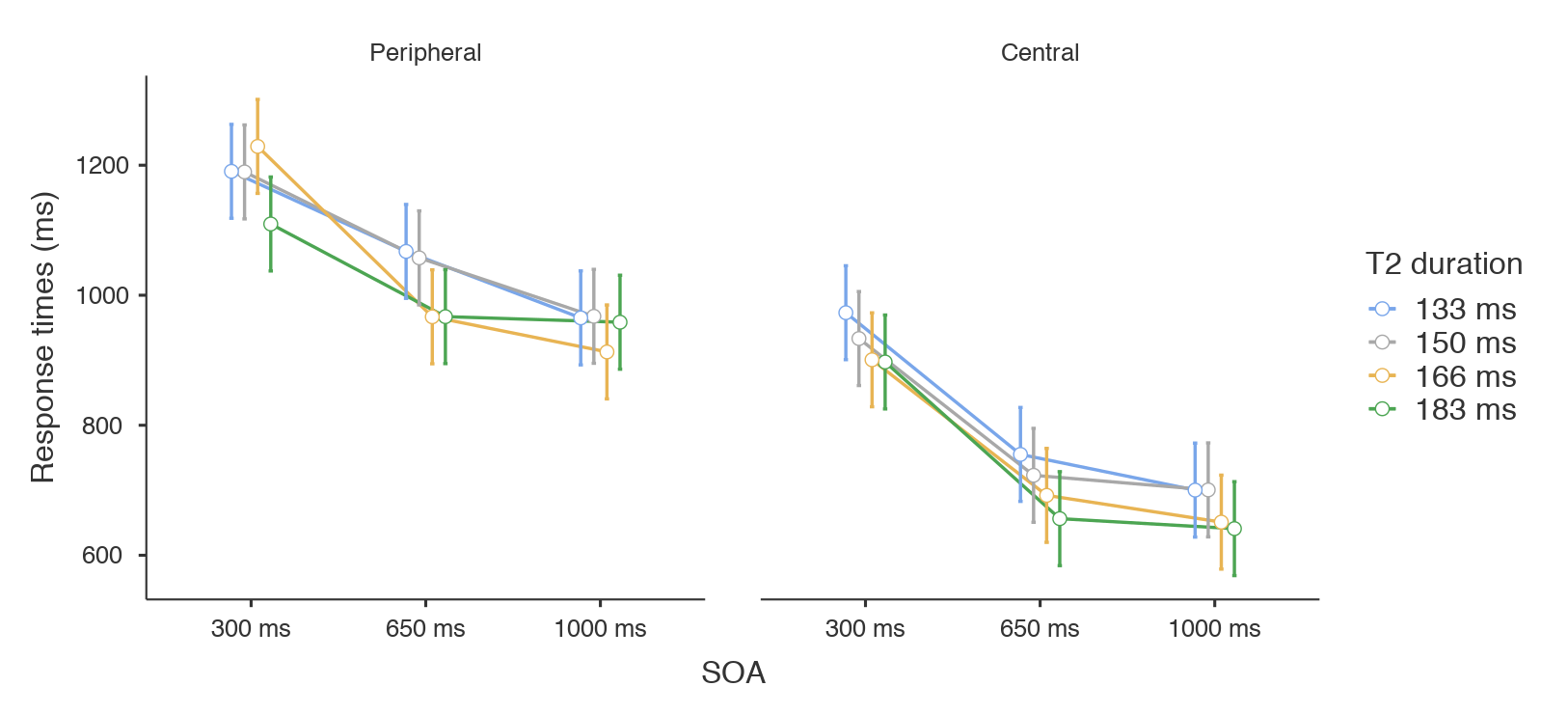


Figure S1. RT2 analysis for Experiment 1. Vertical bars represent the SEM.

***Experiment 2.*** Main effect of SOA, F(1.19, 15.52) = 94.980, p < 0.001, eta = 0.880, with RT2 249 ms slower at shortest vs. longest SOA. Main effect of T2 duration, F(1.4, 18.23) = 6.105, p = 0.016, eta = 0.32, with RT2 28 ms slower at shortest vs. longest T2 presentation duration. SOA X T2 duration X T1 modality triple interaction, F(1.51, 19.69) = 4.749, p = 0.029, eta = 0.268. No other significant effect, all *F*s < 1 (*p*s > 0.5).

Further decomposing the triple interaction with two separate RM ANOVAs reveals there is a marginal SOA X T2 presentation duration interaction in the auditory condition, F(1.89, 24.53) = 2.92, p = 0.076, eta = 0.183, whereas this interaction is significant in the visual T1 condition, F(2.47, 32.08) = 3.66, p = 0.029, eta = 0.219. Specifically, the triple interaction reflects the fact that these two interactions go in opposite directions: In the auditory T1 modality, SOA effect is largest in the shortest (306 ms), compared to the longest (222 ms) T2 duration; in contrast, SOA effect is largest in the longest (296 ms), compared to the shortest (208 ms) T2 duration, in the visual T1 modality.


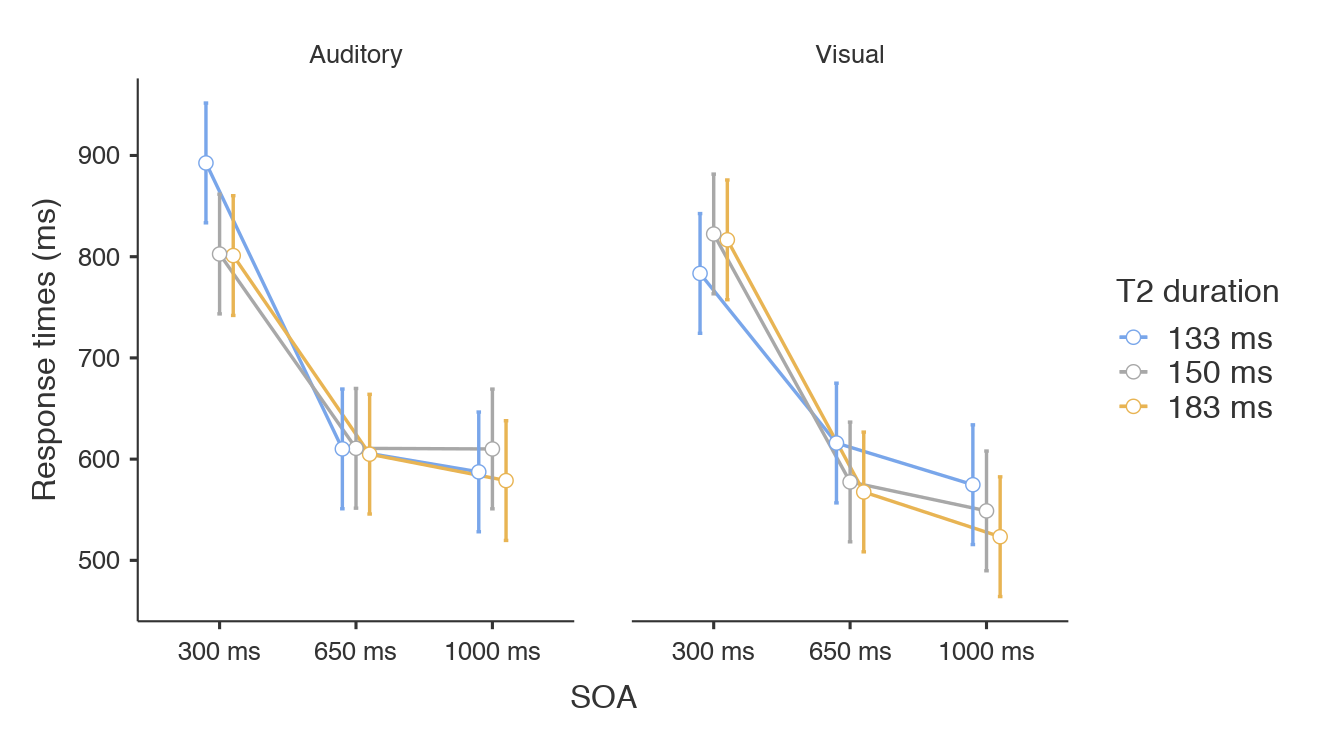


Figure S2. RT2 analysis for Experiment 2. Vertical bars represent the SEM.

***Experiment 3.*** Main effect of T1 type, F(1, 13) = 4.804, p = 0.047, eta = 0.27, with RT2 60 ms slower in the square (no task switch) vs. rectangle width (task switch) condition. Main effect of SOA, F(1.07, 13.93) = 76.493, p < 0.001, eta = 0.855, with RT2 261 ms slower at shortest vs. longest SOA. SOA X T2 duration interaction, F(2.34, 20.48) = 3.261, p = 0.045, eta = 0.201. No other effect reached significance, *F*s < 2.26 (*p*s > 0.14).

The interaction can thus be qualified: SOA effect is largest at intermediate (322 ms), compared to short (225 ms) and long (237 ms) T2 presentation duration. However, the 12 ms (*SE* = 68.5 ms) difference in SOA effect between short and long T2 presentation duration is nonsignificant, t(13) = 0.261, p > 0.7, d = 0.07. Thus, it is unlikely that a speed-accuracy tradeoff would account for differences in accuracy.


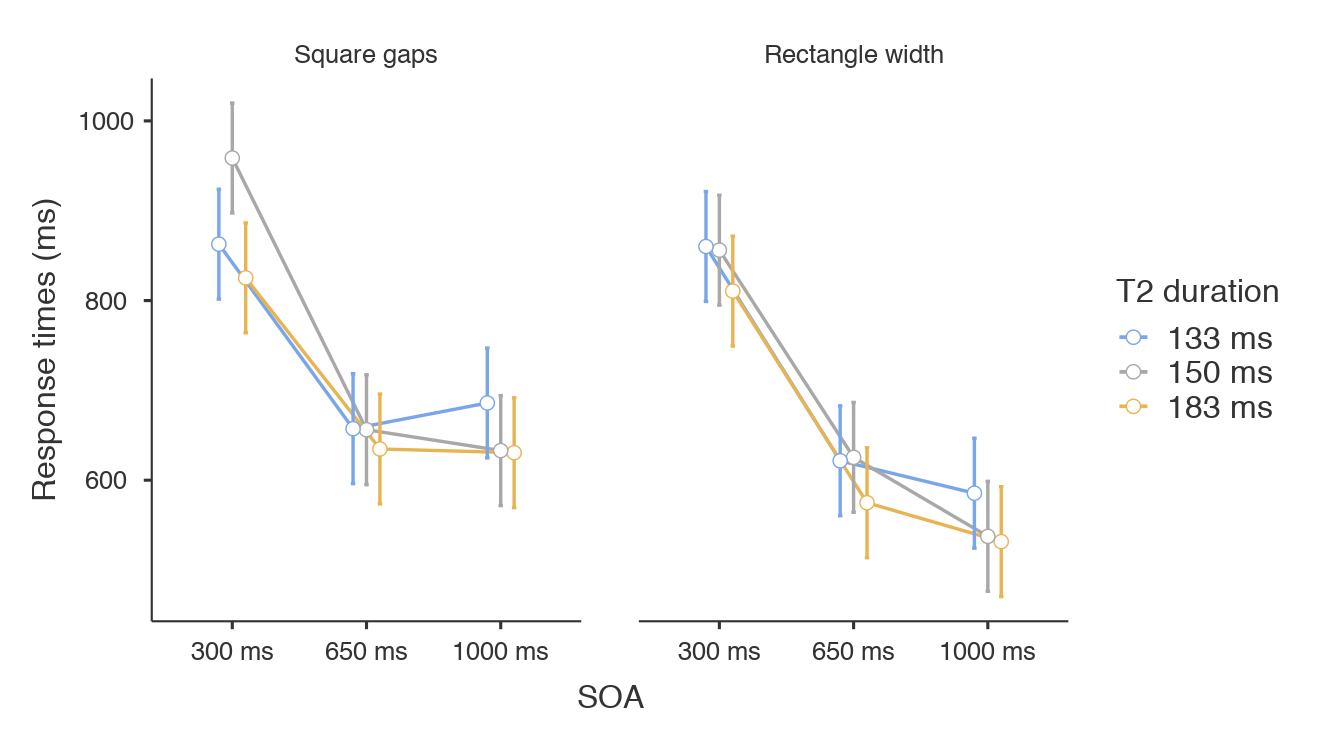


Figure S3. RT2 analysis for Experiment 3. Vertical bars represent the SEM.

***Experiment 4*.** Main effect of response modality, F(1, 11) = 4.339, p = 0.061, eta = 0.283, with RT2 36 ms slower in the hand switching condition, vs. no switch. Main effect of SOA, F(1.33, 14.59) = 106.34, p < 0.001, eta = 0.906, with RT2 150 ms slower at shortest vs. longest SOA. Main effect of T2 presentation duration, F(2, 22) = 26.283, p < 0.001, eta = 0.705, with RT2 50 ms slower at shortest vs. longest T2 presentation duration. Response modality X T2 presentation duration interaction, F(2, 22) = 7.706, p = 0.003, eta = 0.412. No other significant effect, Fs < 2 (ps > 0.13).

The interaction can thus be qualified: The effect of response modality was more modest at short (25 ms), compared to intermediate (42 ms) and long (40 ms) T2 presentation duration. In other words, removing hand switching from the equation accelerated response input to a greater extent for intermediate and long T2 presentation durations, vs. short T2 presentation durations across all SOA conditions.


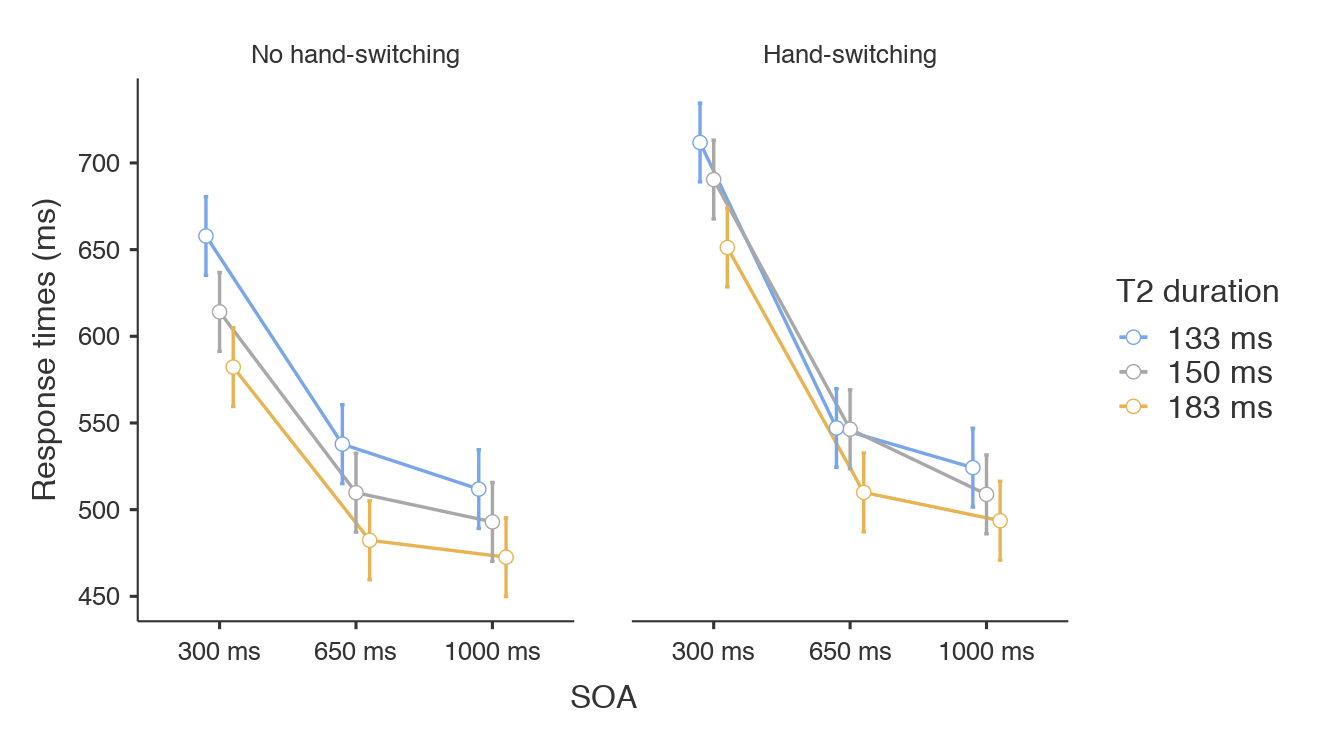


Figure S4. RT2 analysis for Experiment 4. Vertical bars represent the SEM.

***Discussion.*** One possible indicator of a speed accuracy tradeoff which could explain T2|T1 accuracy drops in our experiments would be the presence of an interaction between SOA and whichever potential interference locus was controlled (e.g., in Exp 1, SOA X spatial attention; in Exp. 2, SOA X T1 modality, and so forth). Specifically, a smaller SOA effect in the locus control (e.g., central T2) vs., uncontrolled locus condition (e.g., peripheral T2) would hint at a speed accuracy tradeoff. To this effect, no SOA X interference locus was found.

Another possible indicator of a speed accuracy tradeoff would be a main effect of interference locus, such that RT2 was faster and T2 accuracy was lower in the locus control vs. uncontrolled locus condition. Again, close examination revealed no such pattern. Though there was an effect of spatial attention in Exp. 1, as RT2 was faster in the central vs. peripheral T2 condition, overall accuracy was also higher in the central vs. peripheral condition, which indicates that the peripheral condition was simply more difficult than the peripheral condition, as would be expected. In Exp. 3, overall T2 accuracy was about 5% lower in the square gap (no task switch) vs. rectangle width (switch) T1 condition, but RT2 was also slower, not faster, in the former vs. the latter. Finally, in Exp. 4, RT2 was faster in the no hand switching vs. hand switching condition, but T2 accuracy was also higher in the no hand switching vs. hand switching condition.

There remains the triple SOA X T2 presentation duration X T1 modality interaction found in Exp. 2, which requires more elaboration. Indeed, the typical (at least, in our study) T2 presentation duration effect (longer RT2 and lower T2 accuracy for shorter T2 presentation durations) interacted with SOA when T1 was presented in the visual modality, such that the effect on RT2 was reversed at short SOA. In other words, the SOA effect on RT2 diminished as T2 presentation duration decreased when T1 was also visual, whereas the (marginally significant) opposite pattern was observed when T1 was auditory. As it pertains to T2 accuracy however, there was no such triple interaction, and SOA and T2 presentation interacted in the same way: The SOA effect on T2 accuracy diminished as T2 presentation duration increased, irrespective of T1 modality. That is, effects of SOA and T2 presentation duration on T2 accuracy were similar across T1 modalities. In other words, the “reverse” SOA X T2 interaction effect on RT2 in the visual T1 modality did not translate to T2 accuracy, which would have been evidence of a speed-accuracy tradeoff.

Finally, let us suppose for argument sake that there is in fact a speed accuracy tradeoff at play in Experiment 2. In that case, it should be attributed to the shift from an auditory-visual T1-T2 pair to a visual-visual T1-T2 pair. As such, this speed-accuracy tradeoff should also be apparent in Exp. 3 and 4. As we have already made explicit, this is not the case.

For all these reasons, it seems clear to us that our accuracy results cannot be explained in term of a speed accuracy tradeoff. Accuracy drops at short SOA did not result from a decrease in response speed, but from faulty visual encoding.
